# Argon Ion Treatment of Multi-Material Layered Surface-Electrode Traps for Noise Mitigation

**DOI:** 10.3390/e27121208

**Published:** 2025-11-28

**Authors:** Deviprasath Palani, Florian Hasse, Philip Kiefer, Frederick Böckling, Daniel L. Stick, Dustin Hite, Ulrich Warring, Tobias Schaetz

**Affiliations:** 1 Physikalisches Institut, Albert-Ludwigs-Universität Freiburg, 79104 Freiburg, Germany; 2 Sandia National Laboratories, Albuquerque, NM 87123, USA; 3 National Institute of Standards and Technology, 325 Broadway, Boulder, CO 80305, USA

**Keywords:** argon-ion treatment, anomalous heating, quantum computing, ion traps, surface-electrode traps, noise mitigation

## Abstract

Electric-field noise near ion-trap electrodes limits motional coherence and represents a key obstacle to scaling trapped-ion quantum systems. Here, we investigate how in situ Ar^+^ sputtering modifies motional heating and dephasing in multi-material surface-electrode traps. Trapped ions serve as local probes of electric-field fluctuations before and after controlled sputtering cycles. The data reveal a non-monotonic dependence of both the dephasing rate and the electric-field noise on the extent of Ar^+^ sputtering, with coherence initially improving while heating rates increase, followed by a reversal at longer exposures. This behavior highlights the intricate balance between beneficial surface cleaning and detrimental structural modification, driven by changes in surface morphology, redeposition of sputtered material, and diffusion on the surface, underscoring the complex interplay between surface composition and motional stability in multi-material electrode systems. Post-treatment scanning electron microscopy and energy-dispersive X-ray spectroscopy confirm significant modification of the multilayer structure. Technical noise was independently verified to be well below the observed levels. These findings indicate that in situ sputtering modifies surface properties in ways that can either mitigate or enhance electric-field noise, underscoring the need for precise control of material interfaces in next-generation ion-trap architectures.

## 1. Introduction

Decoherence is a universal challenge across quantum technologies [1,2,3,4,5,6,7,8,9]. Scaling to useful, larger quantum systems can only succeed if this challenge is addressed. Some decoherence mechanisms, such as charge and electric-field noise, appear across multiple platforms and motivate collaborative efforts to identify their microscopic origins and mitigation strategies [3,5,10,11,12,13,14]. In trapped-ion systems, one of the primary decoherence channels arises from motional heating driven by electric-field fluctuations near the electrodes [5,15,16,17]. This phenomenon, commonly referred to as *anomalous heating* because its magnitude exceeds expectations based on known technical noise sources and its microscopic origin remains poorly understood, scales strongly with the ion–electrode distance, approximately following a $1/d^{4}$ dependence [16,18,19]. Despite advances in materials, fabrication, and environmental control, the microscopic mechanisms behind anomalous heating remain unresolved. Experimental studies suggest that this heating originates from surface-induced electric-field noise driven by microscopic fluctuating patch potentials, while theoretical work attributes it to dipolar adsorbates and other thermally activated surface dynamics [17,20,21,22]. However, its quantitative dependence on temperature and material composition remains poorly understood, and even nominally identical traps, fabricated with the same materials and geometries, exhibit heating rates that differ by orders of magnitude, indicating additional contributions from uncontrolled surface conditions such as adsorbates, oxides, or residual fabrication contaminants [23]. Current ion-trap architectures are rapidly evolving towards higher levels of integration, incorporating advanced functionalities such as embedded photonic waveguides [24,25,26,27], integrated detectors [28,29], and on-chip microwave circuitry for coherent control [30,31,32,33]. While these developments expand capability and scalability, they also introduce greater complexity in surface dynamics, material interfaces, and potential noise sources. As trap designs evolve toward increasingly complex multilayer structures with diverse surface materials such as combinations of metals and dielectrics, the interplay of contamination, diffusion on the surface, and surface morphology introduces additional potential sources of noise. Surface treatment methods such as argon ion sputtering, plasma cleaning, and laser cleaning have shown promise in reducing noise in single-material traps [18,34,35,36,37], but their effectiveness and compatibility within complex multi-material architectures have not yet been systematically evaluated and thus warrant closer examination.

In this study, we address this critical gap by evaluating the effectiveness of in situ argon ion surface treatments (sputtering) specifically in multi-material layered traps. We quantify the impact of treatment on heating rates, decoherence of motional quantum states, and surface integrity, providing insights into the compatibility and optimization of these techniques required for advanced and complex trap designs. Employing a combination of precise in situ electric-field measurements using trapped magnesium ions, alongside detailed ex situ surface analysis methods, we characterize both the beneficial and the detrimental effects of argon ion sputtering on multi-material surfaces.

## 2. Materials and Methods

Our experiments are carried out at ambient temperature (exact surface temperature unknown during operation) in a custom ultra-high-vacuum (UHV) chamber equipped with optical viewports and housing multilayer complementary metal-oxide-semiconductor (CMOS)-compatible trap chips [38]. In situ electric-field sensing is performed using trapped Mg+25 ions, where a hyperfine qubit [39,40] probes motional degrees of freedom to access local electric-field noise at distinct motional mode frequencies. The CMOS fabrication process yields a complex stack of aluminum (Al), titanium/platinum (Ti/Pt) diffusion barriers, gold (Au), and dielectric layers, where the top gold film is only tens of nanometer thick, in contrast to single-metal-layer traps that employed several-micrometer-thick gold films on quartz substrates. By interleaving motional heating and motional dephasing measurements with controlled argon ion (Ar^+^) sputtering, we directly treat and characterize surface adsorbates without venting the vacuum system. To isolate surface-induced noise, we benchmark technical contributions from radiofrequency (RF) noise, direct-current (DC) noise filtering, and residual light leakage, confirming that they fall orders of magnitude below the anomalous heating we measure. Prior to the main treatment sequence, we perform ex situ surface analysis via scanning electron microscopy (SEM), light microscopy (LM), and energy-dispersive X-ray spectroscopy (EDX) to map morphological changes, elemental redeposition, and cross-layer diffusion. This combined in situ/ex situ approach provides a comprehensive evaluation of both treatment efficacy and the resulting surface alterations.

### 2.1. Vacuum System and Chip Architecture

**Figure 1:**
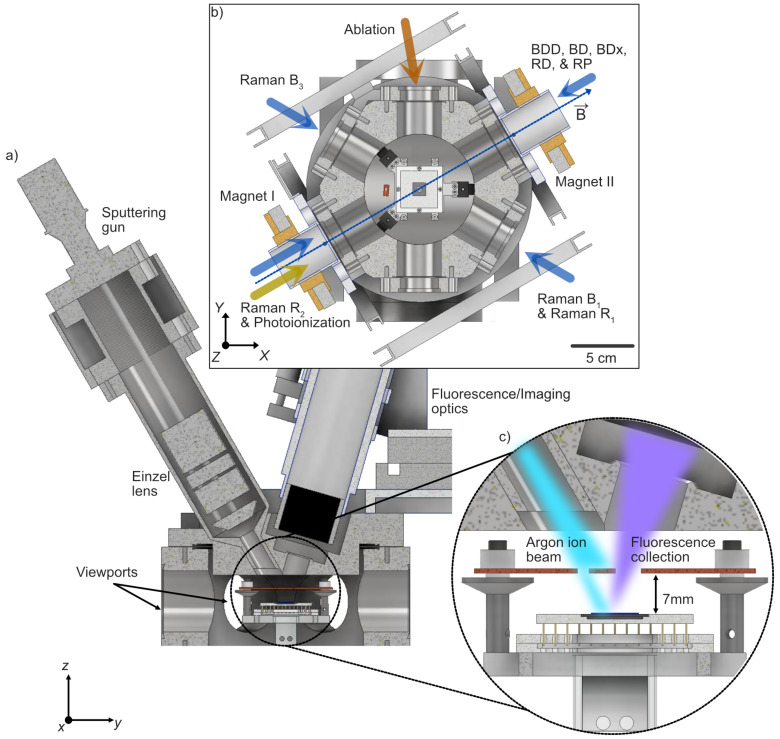
Cross-sectional views of the UHV chamber. (**a**) Side render of the hexagonal chamber showing the 100-pin chip-carrier socket, optical viewports for fluorescence detection, and the argon ion gun mounted at an angle of $\theta_{\mathrm{sputter}}≃30^{\circ }$ relative to the chip surface normal (*z*-axis) and ≃$120^{\circ }$ from the *y*-axis. (**b**) Top view illustrating optical access ports for laser beams and magnetic-field components. Permanent magnets generate a quantization field of $|\vec{B}|\;≃10.95\;\mathrm{mT}$, complemented by coil pairs for fine tuning. Also shown are Doppler cooling beams (BDD, BD, BDx), repumping beams (RD, RP), four Raman beams ($B_{1}$, $R_{1}$, $R_{2}$, $B_{3}$), a photoionization beam, and the ablation laser. (**c**) Close-up cross-section of the trap chip mounted on its CPGA carrier below the gold-plated copper mask ($h_{\mathrm{Mask}}≃7\;\mathrm{mm}$). The mask includes apertures for Ar^+^ beam collimation and fluorescence collection and is electrically connected (not shown) for use as an off-plane electrode to compensate stray fields along the *z*-axis and as a pickup electrode during argon ion sputtering.

The UHV chamber features six anti-reflection (AR) coated viewports for high UV transmission (Figure 1). It is evacuated by an Agilent TwisTorr 304 FS turbomolecular pump, an Agilent VacIon Plus 300 StarCell ion pump, and a titanium sublimation pump (TSP), maintaining a base pressure of $P_{\mathrm{res}}\leq 10^{-8}\;\mathrm{Pa}$, with brief unresolvable spikes observed during ion loading before recovery to baseline. Furthermore, during in situ argon ion sputtering, the argon-dominated partial pressure increases to about $10^{-4}\;\mathrm{Pa}$ (details provided below). After the treatment, the pressure gradually recovers to its baseline value within a few hours. The trap-chip assembly is mounted on a custom socket that holds a ceramic pin-grid-array (CPGA) carrier, providing up to 100 electrical connections to the trap electrodes through vacuum electrical feedthroughs. A gold-plated metallic mask (∼1μm thick) is positioned at $h_{\mathrm{Mask}}≃7\;\mathrm{mm}$ above the trap surface. It includes two apertures, one aligned to the imaging axis and another defining the Ar^+^ beam profile (see Figure 1c), and is electrically connected to a dedicated feedthrough that allows application of a global electric field along the *z* direction. During in situ sputtering, the mask serves both as an electrostatic reference plane that suppresses stray charging and as a pickup electrode to monitor the local ion-beam current. This configuration ensures that any charge accumulation on the mask or trap surface remains minimal and reproducible between treatment cycles. The top flange holds a backfill-type sputtering gun (RBD Instruments 04-165) mounted at an angle of $\theta_{\mathrm{sputter}}≃30^{\circ }$ with respect to the chip surface normal. A dedicated gas inlet line, isolated by a corner valve and pumped by a compact Pfeiffer turbomolecular-pump stand, supplies 99.9999% pure argon through a Pfeiffer UDV 040/140 precision valve on demand. During Ar^+^ sputtering, the argon partial pressure is regulated at $P_{\mathrm{Ar}}≃6\times 10^{-3}\;\mathrm{Pa}$, a value optimized to maintain a stable beam current.

**Figure 2:**
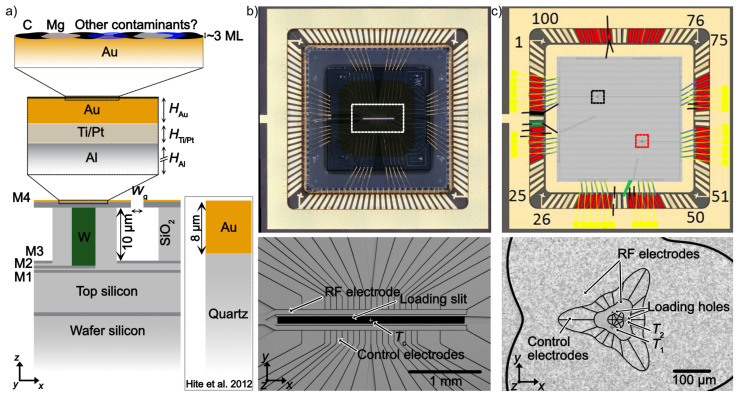
Chip-trap architectures used in this work. Both $10\times 10\;{\mathrm{mm}}^{2}$ chips were fabricated at Sandia National Laboratories using CMOS-compatible multilayer technology. The top metal stack consists of Au, Pt, Ti, and Al. (**a**) Cross-sectional schematic of the multilayer trap architecture, shown alongside a single-metal-layer trap used in earlier Ar^+^ treatment studies [18]. From bottom to top: the silicon substrate supports multiple metal layers (M1 and M2), with vertical tungsten (W) vias providing connectivity to the surface electrodes. An intermediate metal M3 layer shields the ion from exposed dielectrics, while the metal M4 layer includes an Al–0.5%Cu film ($H_{\mathrm{Al}}∼2\;\mu m$), a Ti/Pt diffusion barrier ($H_{\mathrm{Ti}/\mathrm{Pt}}∼50$–60nm), and a final Au coating ($H_{\mathrm{Au}}∼50$–70nm). Surface contaminants such as carbon, magnesium (from ion loading), and other residues can accumulate during fabrication, handling, UHV operation, and repeated loading attempts, up to about ∼3 monolayers (ML). (**b**) Packaged linear trap (Eurotrap) featuring 42 control connections via an interposer chip and a split RF electrode, with a central loading slit for photoionization of neutral atoms. Bottom: SEM image of the central zone with the loading slit. The long RF rails provide radial confinement in the *y*–*z* plane, while segmented DC electrodes confine ions along *x*. This device was used for in situ Ar^+^ sputtering and subsequent ex situ material analysis. (**c**) A triangular-array trap mounted on its carrier and wire-bonded (yellow squares), showing two equilateral lattices with intersite spacings of 40μm (red square) and 80μm (black square), offset by ∼eq3.4mm. Bottom: SEM image of the 40μm array, showing two RF electrodes and 30 control electrodes that form multiple pseudopotential minima. The lowest three minima define an indented triangular site comprising three trapping locations with 40μm spacing and ion heights of 40μm. Additional ancilla sites at varying heights enable flexible multi-ion configurations [41]. This trap was used for in situ Ar^+^ sputtering and electric-field noise characterization.

The two trap chips used in this study were manufactured at Sandia National Laboratories and designed to incorporate miniaturized electrode geometries for robust ion confinement and multidimensional quantum simulations (Figure 2). Both devices measure $10\times 10\;{\mathrm{mm}}^{2}$ and were fabricated on silicon substrates using a multilayer CMOS-compatible process. Each chip is mounted on a $33\times 33\;{\mathrm{mm}}^{2}$ CPGA carrier that provides mechanical stability and up to 100 reliable electrical connections for integration into the UHV system. The layer stack comprises multiple layers of dielectric silicon dioxide (${\mathrm{SiO}}_{2}$), aluminum–0.5% copper (Al–0.5%Cu) electrodes and routing leads, a Ti/Pt diffusion barrier (50–60nm), and a 50–70nm Au top layer (see Figure 2). Inter-electrode gaps of ≃1.3μm and a 10μm elevation above the buried leads help minimize stray electric fields. Surface contaminants such as carbonaceous and oxide films rich in carbon (C) and oxygen (O) species [18] can accumulate during fabrication, handling, or exposure to air, and during UHV operation. Typical fabrication layers and contamination films are illustrated in Figure 2a. These surface films alter the local work function and create microscopic patch potentials that introduce additional electric-field noise, leading to enhanced motional heating and reduced motional coherence. Integrated 820pF trench capacitors are fabricated on the interposer to connect each DC electrode to ground; alternatively, 820pF capacitors on the CPGA carrier provide the same function. In both cases, they suppress pickup of the RF drive and other high-frequency noise above their cut-off frequency, while aluminum wire bonds provide low-resistance electrical contacts. The radio-frequency (RF) drive is generated by a low-noise Rohde & Schwarz SMA100B source operating at $\Omega_{\mathrm{RF}}/2\pi \approx 60\;\mathrm{MHz}$ with an output power of approximately 26dBm. The signal is coupled to a helical resonator mounted outside the vacuum chamber, which provides impedance matching and voltage amplification. This configuration yields a zero-to-peak voltage at the trap electrodes of about $V_{\mathrm{zp}}\approx 200\;V$, consistent with finite-element simulations and the measured secular frequencies. With a loaded quality factor of Q≈100, the resonator rejects RF noise outside a bandwidth of about 600kHz around $\Omega_{\mathrm{RF}}$, thereby filtering broadband fluctuations. The resonator housing is electrically shielded and grounded to minimize environmental pickup.

The Eurotrap chip (Figure 2b) features 42 segmented DC electrodes and a split central RF rail that generate a pseudopotential minimum at $h_{\mathrm{ion}}≃83\;\mu m$ above the surface [42,43]. A central loading slit, 100μm wide and extending over two-thirds of the trap length, enables neutral-atom vapor injection for photoionization. The material stack consists of a 70nm Au top layer, a 60nm Ti/Pt diffusion barrier, a 2μm Al base, and 10μm-deep tungsten vias. Each DC electrode includes on-board capacitive filtering and is additionally connected to an external single-stage resistor-capacitor (RC) filter located outside the vacuum through a hermetic feedthrough, providing further suppression of pickup and broadband electrical noise. The segmented layout allows axial confinement and stray-field compensation via independent control of 42 electrodes [44]. During Ar^+^ sputtering, a ∼4–5mm diameter ion beam is aligned through a 1mm mask aperture positioned $h_{\mathrm{Mask}}≃7\;\mathrm{mm}$ above the surface to achieve localized treatment of the electrode region. This trap chip was dedicated to in situ Ar^+^ treatments and subsequent ex situ material analysis and was not employed for explicit noise-characterization measurements.

The triangular-array chip, developed jointly with NIST and R. Schmied [45,46,47,48], contains two arrays with intersite spacings of 40μm and 80μm at corresponding ion heights of 40μm and 80μm (Figure 2c). The arrays are positioned off-center by approximately 3.4mm from the geometric center of the chip. RF electrodes were optimized using a gapless-approximation algorithm [49], enabling three independently controllable main sites ($T_{0}$, $T_{1}$, and $T_{2}$ in Figure 2c), with most experiments conducted at $T_{2}$. The top metal stack is similar to the Eurotrap design and consists (top to bottom) of approximately 50nm of Au, 50nm of Ti/Pt, 2μm of Al, and 10μm-deep vias. A single low-noise RF source connected to a helical resonator and the RF electrodes generates the trapping landscape [41], creating local minima at the trap sites, while 30 segmented control electrodes provide tunability of the local fields and curvatures at each site [45,47,48]. For Ar^+^ sputtering, the 40μm array was targeted using a 3mm-diameter beam emerging from the ion gun, mounted at an incidence angle of $\theta_{\mathrm{sputter}}\approx 30^{\circ }$ relative to the surface normal. The beam was aligned to the active trapping region, positioned approximately 3.4mm laterally from the geometric center of the chip and 7mm below the mask aperture (x,y,z≃2.4,−3.4,7mm). In this configuration, the ion flux illuminated the trapping zone uniformly without striking the neighboring electrodes or the chip edge.

### 2.2. Electric-Field Sensing with Mg Ions

We trap Mg+25 ions (I=5/2) and exploit the hyperfine qubit transitions between S1/22,F=3,mF=+3 (|↓〉) and $F=2,\;m_{F}=+2$ (|↑〉) for high-precision electric-field sensing [39]. Ions are loaded via ablation-assisted photoionization of a magnesium target using a focused pulsed infrared laser with wavelength $\lambda_{\mathrm{abl}}\approx 1030\;\mathrm{nm}$, maximum repetition rate $\nu_{\mathrm{rep}}\approx 1.5\;\mathrm{kHz}$, and pulse energy $E_{\mathrm{abl}}\approx 30\;\mu J$ (5–10 pulses per attempt) [41]. The two-photon ionization sequence drives S01↔P11 at $\lambda_{\mathrm{PI}}=285\;\mathrm{nm}$, followed by a second photon from the 285nm beam or Doppler beam. In the case of the triangular array, trapped ions are shuttled from a loading hub to experimental zones via tailored control waveforms, achieving >90% loading success within a few seconds [41]. We use single trapped Mg+25 ions as local probes of electric-field fluctuations. We distinguish between anomalous noise of unknown surface origin and technical noise from instrumental sources [50]. The anomalous component, often referred to as surface-induced noise, may originate from microscopic variations in the electric field near the electrodes, potentially involving fluctuating patch potentials, and/or other thermally activated processes [17,20,21,22,23,51]. This noise produces motional heating rates that exceed the fundamental Johnson-Nyquist predictions and generally scale with ion-surface distance *h* as electric-field noise spectral density $S_{E}\propto h^{-\beta }$, where $S_{E}\left(\omega \right)$ has units of ${\left(V/m\right)}^{2}/\mathrm{Hz}$ and β≃4 for typical surface-induced noise [17,19,52]. In contrast, technical noise of electronic origin, such as voltage supply fluctuations and ambient electromagnetic pickup, scales more weakly with distance, with β≃2, and defines the circuit-limited background noise floor [19]. We further consider residual light leakage from all laser beams as a potential source of heating mechanism.

Quantum sensing protocols typically proceed through three stages: initialization, manipulation, and detection, a common structure across quantum platforms, including trapped-ion systems [53]. Here, initialization employs Doppler cooling on the S1/22,F=3→P3/22,F=4 transition using a far-detuned beam (BDD) detuned by ≈8Γ and a near-resonant beam detuned by Γ/2 (BD), where Γ/2π≃41.4MHz is the natural linewidth of the transition. Repumping employs the red Doppler (RD) and repumper (RP) beams, both coupling to the P1/22,F=2 manifold to depopulate all other hyperfine sublevels of the S1/22,F=3 state and prepare the |↓〉 state with high fidelity. Coherent manipulation uses a global microwave drive near $\Omega_{\mathrm{MW}}/2\pi ≃1.8\;\mathrm{GHz}$ and two-photon stimulated Raman transitions detuned by $\Delta_{\mathrm{TPSRT}}/2\pi ≃20\;\mathrm{GHz}$ (Lamb–Dicke parameter η≈0.1) [54]. Detection uses near-resonant Doppler (BDx) illumination on |↓〉↔P3/22,F=4 (bright) versus |↑〉 (dark). Photon counts collected by a photomultiplier tube (PMT) follow Poissonian statistics. From these distributions, population probabilities are estimated, and the corresponding heating rates are extracted.

We employ three complementary techniques to measure the ion’s motional energy distribution (Table 1). These methods span dynamic ranges from $\bar{n}≳100$ down to $\bar{n}\ll 1$, with varying requirements for state preparation, where $\bar{n}$ denotes mean motional quanta.

The first method, for low motional excitation ($\bar{n}<3$), we performresolved sideband thermometry on motional modes in the range $\omega_{i}/2\pi =1–6\;\mathrm{MHz}$, where $\omega_{i}$ denotes the frequency of the *i*-th motional mode and the corresponding Lamb–Dicke parameters are $\eta_{i}=0.1–0.3$. Doppler cooling reduces the thermal occupation to ${\bar{n}}_{\mathrm{th}}\leq 10$, followed by pulsed Raman sideband cooling to reach near-ground-state populations. The mean motional excitation $\bar{n}$ is determined from the excitation probabilities on the first-order red $P_{\mathrm{red}}$ and blue sidebands $P_{\mathrm{blue}}$, which scale as shown in Equation (1).

(1) $$P_{\mathrm{blue}}\propto \bar{n}+1,\;P_{\mathrm{red}}\propto \bar{n}$$

The relative contrast $C_{\mathrm{SB}}$ between these sidebands provides a direct measure of $\bar{n}$, as expressed in Equation (2).

(2) $$C_{\mathrm{SB}}\left(\bar{n}\right)=\frac{P_{\mathrm{blue}}-P_{\mathrm{red}}}{P_{\mathrm{blue}}+P_{\mathrm{red}}}=\frac{1}{2\bar{n}+1}$$

The sensitivity of this contrast to changes in motional excitation is given by Equation (3), which highlights sub-phonon resolution for $\bar{n}<1$.

(3) $$\frac{dC_{\mathrm{SB}}}{d\bar{n}}=-\frac{2}{{\left(2\bar{n}+1\right)}^{2}}$$

To extract heating rates $\dot{\bar{n}}$, we introduce a controlled delay between cooling and probing and evaluate the corresponding electric-field noise spectral density using Equation (4) [17], where *e* is the elementary charge, *m* the ion mass, *ℏ* the reduced Planck constant, and ω the motional frequency.

(4) $$\dot{\bar{n}}=\frac{e^{2}}{4m\hbar \omega }\;S_{E}\left(\omega \right)$$

The next method, the carrier method, provides a sensitive probe of intermediate motional excitation by exploiting the dependence of Rabi oscillations on the motional state during the spin-flip (carrier) transition, implemented via a resonant two-photon stimulated Raman process [55]. After Doppler and optional sideband cooling, the ion is initialized in the |↓〉 state and coherently driven on the carrier transition |↓,n〉↔|↑,n〉 using a resonant two-photon Raman interaction. The bright-state population then evolves as given in Equation (5).

(5) $$P_{↓}\left(t\right)=\frac{1}{2}\left[1+\sum_{n=0}^{\infty }P_{n}\;e^{-\gamma_{n}t}\cos\left(2\Omega_{n}t\right)\right]$$

In this expression, $P_{n}$ is the population of the motional Fock state *n*, $\gamma_{n}$ is the corresponding decoherence rate, and $\Omega_{n}$ is the motional-state–dependent Rabi frequency. The dependence of $\Omega_{n}$ on the motional quantum number arises from the Lamb–Dicke coupling and follows the generalized Laguerre scaling, as shown in Equation (6).

(6) $$\Omega_{n}=\Omega_{0}\;e^{-\eta^{2}/2}L_{n}^{\left(0\right)}\left(\eta^{2}\right)$$

Here, $\Omega_{0}$ is the carrier Rabi frequency for the ground motional state, η is the Lamb–Dicke parameter, and $L_{n}^{\left(0\right)}$ denotes the generalized Laguerre polynomial. By fitting the measured dynamics from Equation (5) to a thermal or coherently displaced $P_{n}$ distribution, we extract the mean motional excitation $\bar{n}$. To quantify deviations from the ideal ground-state evolution, we define a contrast function as given by Equation (7).

(7) $$C_{↓}\left(t;\bar{n}\right)=P_{↓}\left(t;\bar{n}\right)-P_{↓}^{n=0}\left(t\right)$$

In Equation (7), $P_{↓}^{n=0}\left(t\right)$ represents the carrier evolution for a motional ground state. The sensitivity of this contrast to motional excitation is expressed by Equation (8).

(8) $$\frac{\partial C_{↓}\left(t;\bar{n}\right)}{\partial \bar{n}}=\frac{1}{2}\;e^{-\gamma t}\sum_{n=0}^{\infty }\frac{\partial P_{n}}{\partial \bar{n}}\cos\left(2\Omega_{n}\;t\right)$$

Equation (8) quantifies the response of the carrier contrast to variations in $\bar{n}$, establishing the achievable resolution of the method. This approach provides moderate sensitivity over a broad dynamic range, typically achieving $\Delta \bar{n}≳3$.

The last method, the Doppler method, probes large motional amplitudes by monitoring the reduction in Doppler-sensitive fluorescence [44,46]. Coherent or incoherent excitation of the motional mode broadens the ion’s velocity distribution and thereby decreases resonant scattering, leading to a measurable drop in fluorescence. The fluorescence level $R^{\mathrm{fl}}$ decreases approximately exponentially with the mean motional excitation $\bar{n}$, as expressed in Equation (9).

(9) $$R^{\mathrm{fl}}=R_{0}^{\mathrm{fl}}\;\exp\left(-k\;\bar{n}\right)$$

Here, $F_{0}$ is the fluorescence rate when all motional modes are cooled near the ground state, and *k* is an empirical calibration constant (in units of phonon^−1^) that incorporates the effects of mode frequency, laser geometry, and optical linewidth. This relation enables a direct estimate of $\bar{n}$ from the measured fluorescence reduction and is particularly effective for highly excited states ($\bar{n}≳100$), where sideband-based thermometry becomes insensitive. To probe motional coherence limits arising from trap-frequency fluctuations and ambient electric-field noise, we apply a Ramsey-like displacement sequence. After Doppler cooling, the ion is displaced by applying a sinusoidal voltage to a control electrode at the motional mode frequency $\omega_{\mathrm{exc}}=\omega_{i}$, as defined in Equation (10), where $U_{\mathrm{exc}}$ is the drive amplitude and $\phi_{\mathrm{exc}}$ is the phase.

(10) $$U\left(t\right)=U_{\mathrm{exc}}\;\cos\left(\omega_{\mathrm{exc}}t+\phi_{\mathrm{exc}}\right)$$

This drive implements the displacement operator D(α), defined in Equation (11), with complex amplitude α determined by $U_{\mathrm{exc}}$ and $\phi_{\mathrm{exc}}$.

(11) $$D\left(\alpha \right)=\exp\left(\alpha a^{†}-\alpha^{*}a\right)$$

Here, $a^{†}$ and *a* denote the creation and annihilation operators of the harmonic oscillator. After a variable wait time $t_{\mathrm{wait}}$, a second displacement of equal amplitude is applied with a relative phase Δϕ. For $\Delta \phi =0^{\circ }$, the two displacements add coherently, whereas for $\Delta \phi =180^{\circ }$, they cancel, ideally returning the ion to its initial state. Scanning Δϕ yields interference fringes whose contrast decays with increasing $t_{\mathrm{wait}}$, defining the motional coherence time or the dephasing rate $\Gamma_{i}$ of the *i*th motional mode. This approach directly probes the phase stability of the motional state without requiring numerical conversion of fluorescence into $\bar{n}$.

Uncertainties in heating and dephasing rates are obtained directly from fits to the experimental data. Heating rates $\dot{\bar{n}}$ are extracted from linear fits to the mean motional excitation $\bar{n}\left(t_{\mathrm{delay}}\right)$, with the 68% confidence interval derived from the fit covariance matrix. Dephasing rates $\Gamma_{i}$ are obtained from exponential fits to the Ramsey-like contrast decay as a function of $t_{\mathrm{wait}}$. Systematic effects, including laser intensity fluctuations, motional-mode drifts, and state-preparation errors, are not plotted separately but are reflected in the scatter of repeated measurements and the fit residuals.

### 2.3. Estimates of Technical Noise Limits

Technical noise sets a lower bound on ion motional heating and must be quantified to isolate surface-induced effects. We evaluate three distinct technical noise channels, RF drive noise, DC control-electrode noise, and residual laser leakage, in dedicated sections below. For each channel, we summarize the noise source, experimental protocol, analysis method, and extracted heating rate. The results show that each technical noise contribution is orders of magnitude below the anomalous heating observed, indicating that surface adsorbent dynamics dominate the overall noise budget.

**Figure 3:**
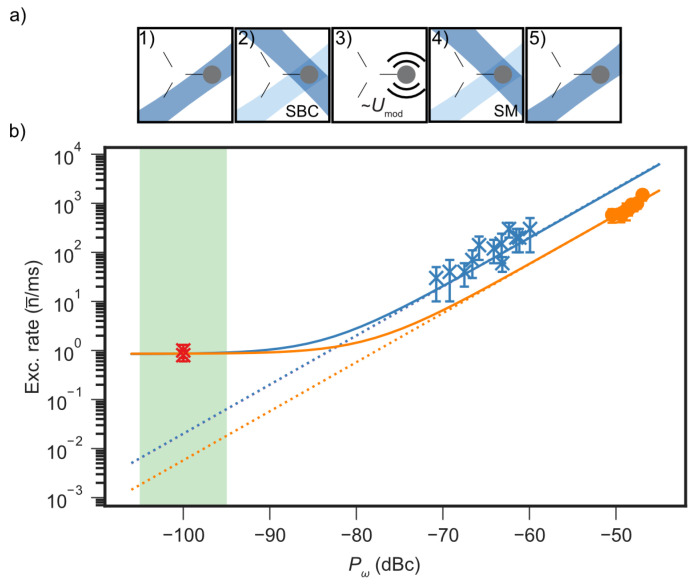
Estimation of RF-noise–induced heating background in the triangular array. (**a**) Experimental sequence: (1) A single ^25^Mg^+^ ion is initialized near the Doppler limit at the $T_{2}$ site. (2) The in-plane mode at $\omega_{1}/2\pi \approx 3.7\;\mathrm{MHz}$ is sideband cooled (SBC) near the ground state using a Raman beam pair ($B_{3}$ and $R_{2}$). (3) The RF drive is frequency-modulated near $\omega_{1}$ for a duration $t_{\mathrm{mod}}∼eq10\;\mu s$, with variable modulation amplitude $U_{\mathrm{mod}}$. (4) Motional excitation is quantified via spin-motion (SM) coupling using sideband contrast thermometry (for low excitation) and carrier methods (for high excitation). (5) Fluorescence detection is used to extract motional populations. (**b**) Measured excitation as a function of the effective modulation level $P_{\omega }$, expressed in dBc relative to the main RF drive and mapped from the applied modulation amplitude $U_{\mathrm{mod}}$. The observed quadratic dependence $\bar{n}\propto P_{\omega }^{2}$ shows coherent excitation driven by the applied signal at the mode frequency. The ambient heating rate (red) is shown at the noise floor (−100±5)dBc, with the uncertainty shaded in green. Quadratic fits (solid: with offset, dotted: without) yield conservative upper bounds of <0.01quanta/ms (blue: sideband) and ∼0.001quanta/ms (orange: carrier) for the technical noise floor, currently limited by the gradient minimization limit, while the offset represents an anomalous heating rate of ∼1quanta/ms. The absence of a resolvable response below this level indicates that stochastic voltage noise contributes only to the root mean square (RMS) background already captured by this bound.

Voltage noise on the RF electrode can couple to the ion motion through micromotion sidebands, leading to additional heating if the ion is displaced from the RF-null point [17,56]. To assess this potential contribution, we characterize the coupling between RF voltage fluctuations and motional excitation by coherently driving a controlled frequency modulation on the RF electrode near the ion’s motional mode frequency. The resulting excitation is quantified using both sideband thermometry and the carrier method, as shown in Figure 3. The feedthrough and wiring present a total series impedance below 20Ω, contributing an electric-potential noise spectral density of $S_{V}≃10^{-17}\;V^{2}/\mathrm{Hz}$, consistent with the Johnson–Nyquist limit for the circuit components. Under the micromotion-minimized configuration, we modulate the RF drive at the secular frequency $\omega_{i}/2\pi =3.7\;\mathrm{MHz}$ using a sinusoidal signal from an arbitrary waveform generator (AWG). The modulation amplitude $U_{\mathrm{mod}}\in \left[0,50\right]\;{\mathrm{mV}}_{\mathrm{RMS}}$ is applied for a duration $t_{\mathrm{mod}}=10\;\mu s$ after preparing the ion near its motional ground state, effectively creating controlled coherent excitations. Calibration measurements show that $U_{\mathrm{mod}}=50\;\mathrm{mV}$ produces first-order modulation sidebands at approximately −50dBc relative to the carrier. Phonon occupation extracted from sideband thermometry and carrier-transition measurements confirms the expected quadratic dependence on modulation amplitude. The sideband method yields a coupling slope of $14\left(2\right)\times 10^{3}\;\mathrm{quanta}/\mathrm{ms}/{\mathrm{dBc}}^{2}$, while the carrier method gives a slightly smaller slope of $7.6\left(5\right)\times 10^{3}\;\mathrm{quanta}/\mathrm{ms}/{\mathrm{dBc}}^{2}$, likely limited by reduced sensitivity to higher excitations and weak anharmonicities. A conservative upper bound from sideband thermometry gives an excitation rate of ${\dot{\bar{n}}}_{\mathrm{RF}}≲0.01\;\mathrm{quanta}/\mathrm{ms}$, whereas the carrier method estimates ${\dot{\bar{n}}}_{\mathrm{RF}}≃0.001\;\mathrm{quanta}/\mathrm{ms}$, both approximately close to the Johnson–Nyquist limit.

Compared to the reference ambient heating rate ${\dot{\bar{n}}}_{\mathrm{amb}}≃1\;\mathrm{quanta}/\mathrm{ms}$ (measured without any additional modulation), plotted at the technical noise floor of −100dBc in Figure 3, we conclude that the heating contribution from technical RF noise is at least two to three orders of magnitude smaller than the anomalous heating observed. The technical noise level must therefore be lower than the bound inferred from coherent excitation, where the resolvable driven response defines the maximum detectable heating rate. Any unresolvable stochastic fluctuations from electronic sources, averaged over the measurement bandwidth, contribute only to the Root Mean Square (RMS) voltage noise already included in this bound, making it a conservative upper limit on the technical heating rate. These measurements demonstrate that the experiment operates at the technical noise floor and that the dominant contribution to the residual heating arises from anomalous, surface-induced electric-field noise rather than from any RF source or its peripherals.

**Figure 4:**
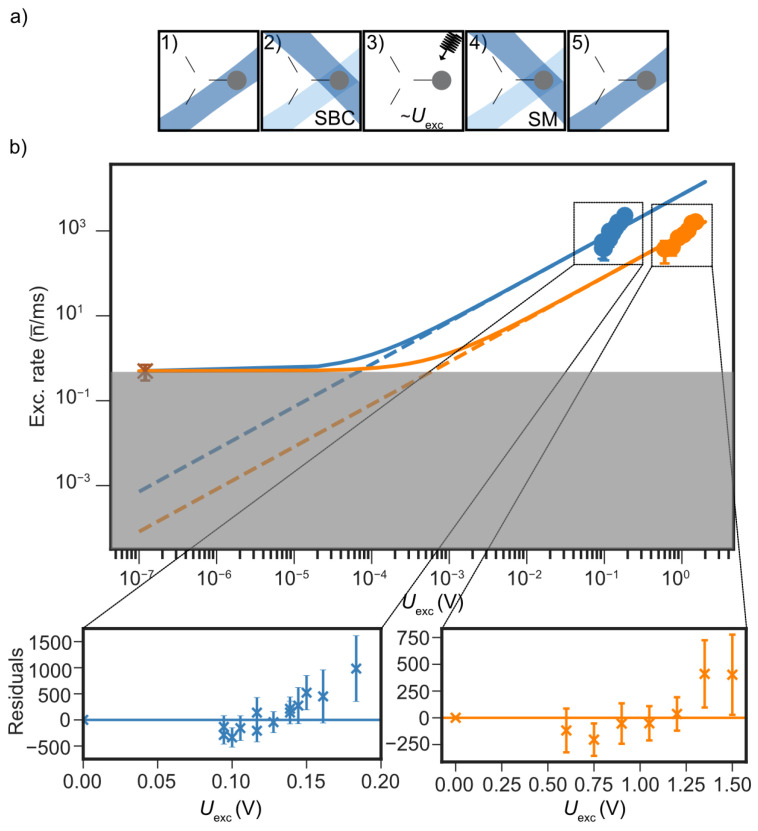
Estimation of DC-noise–induced heating background in the triangular array. (**a**) Experimental sequence: (1) A single ^25^Mg^+^ ion is initialized at the $T_{2}$ site. (2) The in-plane motional mode at $\omega_{1}/2\pi \approx 3.5\;\mathrm{MHz}$ (η≈0.2) is cooled near the ground state using sideband cooling (SBC) with Raman beams ($B_{3}$ and $R_{2}$). (3) Coherent excitation is applied via a control electrode using a single AWG channel with two filter configurations: Set 1 (blue) and Set 2 (orange), while scanning the signal amplitude $U_{\mathrm{exc}}$. (4) Motional excitation is measured via spin-motion (SM) coupling using the carrier method. (5) Readout is performed via fluorescence detection. (**b**) Estimated mean motional excitation as a function of $U_{\mathrm{exc}}$ for both filter sets. The reference heating rate of 1quanta/ms, corresponding to an electric-field noise level of $120\;\mathrm{nV}/\sqrt{\mathrm{Hz}}$, and the estimated noise floor (gray) are indicated. The quadratic dependence $\bar{n}\propto U_{\mathrm{exc}}^{2}$ confirms coherent excitation, with Set 2 showing ≃20dBv suppression relative to Set 1. Both filters approach the Johnson–Nyquist noise limit of ∼0.001quanta/ms, over three orders of magnitude below the ambient heating rate. Residuals at higher excitation reflect systematic effects such as spin-state readout infidelity and weak motional anharmonicity.

Similarly to the RF channel, voltage noise on control electrodes can couple to the ion’s motional mode and lead to heating. To characterize this coupling, we apply a coherent oscillating potential at the secular frequency through a selected control electrode using a channel of the AWG, as shown in Figure 4. Two external first-order low-pass filter configurations were tested: Set 1 (R=26kΩ, C=1nF) and Set 2 (R=512kΩ, C=1nF). Including vacuum feedthroughs and in-vacuum wiring, both configurations were modeled in SPICE as lumped-element networks [57]. At the motional frequency $\omega_{i}/2\pi ≃5\;\mathrm{MHz}$, the expected attenuation is approximately 55dB for Set 1 and 75dB for Set 2. With a near-ground-state-cooled ion, we apply an oscillating potential at $\omega_{i}/2\pi ≃3.5\;\mathrm{MHz}$ for a fixed duration of $t_{\mathrm{mod}}=10\;\mu s$ and vary the drive amplitude. The resulting motional excitation is extracted using the carrier method. As expected for coherent displacement, the excitation scales quadratically with the applied voltage amplitude. A quadratic fit of $\bar{n}$ versus the drive amplitude reveals a relative suppression of about 20dBv when using Set 2 compared to Set 1. Although minor deviations are notable in the data, the fit converges consistently and reproduces the expected quadratic trend. The fit residuals primarily reflect systematic uncertainties arising from spin-state detection infidelity and trap anharmonicities. In Figure 4, two reference points corresponding to the estimated voltage noise amplitude spectral density $S_{V}^{\frac{1}{2}}∼120\;\mathrm{nV}/\sqrt{\mathrm{Hz}}$ mark the ambient heating level [58]. Both filter configurations show quadratic trends that remain well below this reference, with fitted intercepts around 0.001quanta/ms. This offset corresponds to the effective technical noise floor of the DC control electronics and sets a conservative upper bound on the corresponding heating rate inferred from coherent excitation measurements. Using the Equation (4), the inferred electric-field noise spectral density is $S_{E}≲10^{-14}\;{\left(V/m\right)}^{2}/\mathrm{Hz}$ for Set 1, with slightly lower values for Set 2. These measurements indicate that, even when accounting for residual offsets, the technical contribution from the DC control electrodes remains several orders of magnitude below the anomalous heating level.

**Figure 5:**
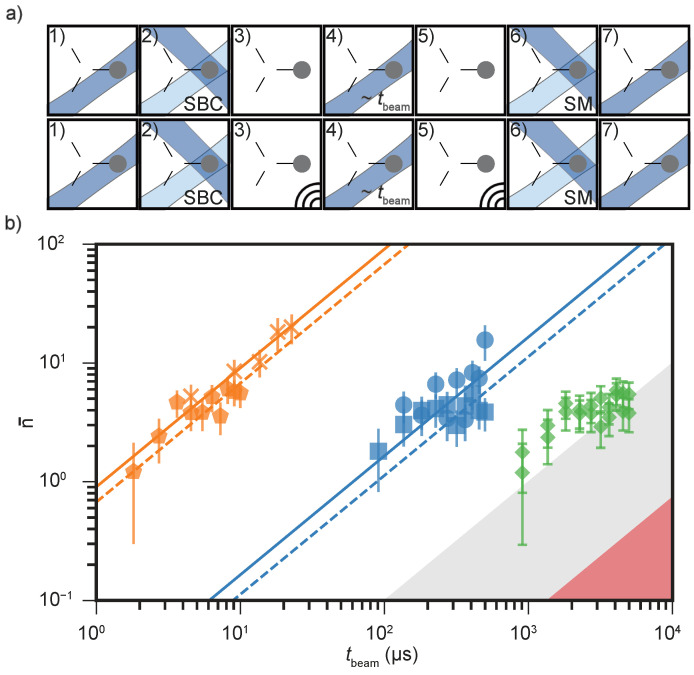
Induced motional excitation due to interaction with laser beams in the triangular array. (**a**) Experimental sequence: (1) A single ^25^Mg^+^ ion is initialized at site $T_{2}$ and Doppler cooled. (2) The in-plane motional mode at $\omega_{2}/2\pi \approx 3.35\;\mathrm{MHz}$ (η≈0.2 for all beams) is sideband cooled (SBC) using Raman beams ($B_{1}$, $R_{2}$). (3) The ion is prepared either in |↓〉 or |↑〉 before each beam exposure. (4) The ion is illuminated by near-resonant BDx and BD beams and by off-resonant Raman, RD, and RP beams for variable durations $t_{\mathrm{beam}}$. (5) The population is returned to |↓〉 with a microwave pulse, and (6) motional excitation is extracted using the carrier method followed by (7) fluorescence detection. (**b**) Mean motional excitation as a function of $t_{\mathrm{beam}}$. Colored markers indicate data for different beams: BDx (orange crosses, blue disks) and BD (orange pentagons, blue squares) produce strong heating in |↓〉, while excitation in |↑〉 is suppressed by about 20dB. Off-resonant Raman, RD, and RP beams (green diamonds) yield heating consistent with the ambient background (gray). An AOM extinction ratio of ≥40dB is assumed for BDx, setting an upper bound (red) on residual heating of ≤0.05quanta/ms.

Residual light from imperfect acousto-optic modulator (AOM) extinction can induce motional excitation through photon scattering. To quantify this effect, we expose a near-ground-state–cooled ion, prepared in either |↓〉 or |↑〉, to individual laser beams (BDx, BD, Raman, RD, and RP) at their nominal detunings and intensities (represented in saturation intensity $I_{\mathrm{sat}}$) for variable durations $t_{\mathrm{beam}}$, as illustrated in Figure 5. The resulting motional heating dynamics follow the rate equations [54] given in Equation (12).

(12) $$A_{\pm }=\eta^{2}\left[W\left(\Delta \right)+W\left(\Delta \mp \omega_{i}\right)\right],\;\dot{\bar{n}}=-\left(A_{-}-A_{+}\right)\;\bar{n}+A_{+}$$

Here, η is the Lamb–Dicke parameter (dimensionless), W(Δ) is the photon-scattering rate at detuning Δ (in s^−1^), and $\omega_{i}/2\pi$ is the frequency of the *i*th motional mode (in Hz). $A_{+}$ and $A_{-}$ represent the transition rates for motional excitation and de-excitation, respectively, both expressed in s^−1^. The quantity $\dot{\bar{n}}$ denotes the net heating rate (in quanta/s), and $\bar{n}$ is the mean motional occupation number. Equation (12) thus captures the balance between heating and cooling processes driven by residual photon scattering from each beam.

Exposure to the BDx beam leads to strong spin-dependent heating via near-resonant photon scattering, with measured rates consistent with theoretical estimates, as summarized in Table 2. Preparing the ion in |↑〉 instead of |↓〉 yields a suppression of approximately 20dB in the heating rate $\dot{\bar{n}}$, indicating that polarization impurities drive off-resonant scattering even in the nominally dark state. The BD beam, detuned by Δ/2π=20MHz, produces reduced heating compared to BDx but still exceeds the ambient level for |↓〉, though it remains below the Doppler limit. Despite their high intensities (∼$300\;I_{\mathrm{sat}}$), the Raman beams detuned by Δ/2π∼20GHz induce heating consistent with the ambient background rate.

We further examined interactions with the BDD, RD, and RP beams, all of which are far detuned from the cooling transition. The BDD (Δ/2π∼320MHz, $I∼50\;I_{\mathrm{sat}}$) can drive weak off-resonant scattering, spin decoherence, and population leakage into other hyperfine states, whereas RD and RP beams contribute heating consistent with the ambient level. Assuming a single-pass AOM extinction ratio of ≥40dB for the BDx, we set an upper bound on residual heating of $\dot{\bar{n}}≲0.05\;\mathrm{quanta}/\mathrm{ms}$, corresponding to an electric-field noise density of $S_{E}≃10^{-13}\;{\left(V/m\right)}^{2}/\mathrm{Hz}$.

We observe ion loss from residual-gas collisions roughly once every 20–30 min at our background pressure $P_{\mathrm{res}}$. These rare losses are attributable to high-energy Langevin-type collisions. Because (i) the trap’s potential retains most energetic collisions and (ii) laser cooling is active during the majority of the experimental duty cycle; only an estimated 1–10% of such events actually remove the ion, while the rest merely induce large but recoverable motional excursions. Glancing collisions, large-impact-parameter fly bys that impart sub-mK energies, occur up to one or two orders of magnitude more frequently under the same conditions [59]. Although we treat these sub-mK “fly by” kicks as negligible in the present heating budget, their cumulative impact remains essentially uncharted. A quantitative study is experimentally demanding because each event is stochastic (Poissonian timing), rare, and minuscule (typically $\Delta \bar{n}\;<\;1$ phonon), three attributes that bury the signal beneath technical noise and laser-cooling transients. Detecting them therefore requires protocols capable of resolving single-phonon displacements over multi-hour data sets.

### 2.4. Argon Ion Treatment Process

Argon sputtering involves bombarding the trap-chip surface with energetic Ar^+^ ions to remove contaminants, modify surface morphology, and etch material layers. In our implementation, we aim to balance cleaning efficacy against risks of material redeposition, electrical shorts, and structural damage [60]. Here, cleaning refers to the removal of surface contaminants and adsorbates on gold electrodes. The treatment was applied on two representative trap architectures, a material study on the Eurotrap (see Appendix A) and the main investigation on the triangular-array trap. Each trap was sputtered multiple times, interleaved with Mg^+^-based performance checks. Key process parameters are summarized in Table 3. Here, $V_{\mathrm{acc}}$ denotes the acceleration voltage applied to generate the Ar^+^ beam, $I_{\mathrm{fil}}$ is the filament current controlling ion emission, $V_{\mathrm{focus}}$ is the focusing voltage that shapes the ion-beam profile, and $T_{\mathrm{treat}}$ is the duration of each sputtering session. Both traps were operated at comparable argon pressures during treatment.

Shielding masks define the ion-beam aperture and optical access region, equivalent to the fluorescence imaging path (see Figure 1). For the Eurotrap, a 1mm-diameter aperture channels the argon beam, delivering $J_{\mathrm{Ar}}≃5\;\mu A/{\mathrm{cm}}^{2}$ to the central electrode region. For the triangular array, a 3mm-diameter aperture yields $J_{\mathrm{Ar}}≃0.35\;\mu A/{\mathrm{cm}}^{2}$. Material removal rates follow from sputter yields and current density (Table 4).

Extended Ar^+^ exposure can cause unintended layer removal and electrical shorts. Assuming $J_{\mathrm{Ar}}=5\;\mu A/{\mathrm{cm}}^{2}$, the estimated etch rates in nm/s and estimated time to remove all the material are tabulated in Table 5.

Across ∼1000min of total exposure, up to 520nm of Al may be removed, occasionally exposing the dielectric layer or damaging RF electrodes. In situ monitoring revealed gold and copper redeposition, and localized electrode damage. Affected electrodes exhibited resistances of $R_{\mathrm{el}}∼0.1$–1MΩ, consistent with partial electrical leakage. Further post-treatment structural and compositional analyses, including SEM and EDX characterization, are provided in Appendix A.

## 3. Results

**Figure 6:**
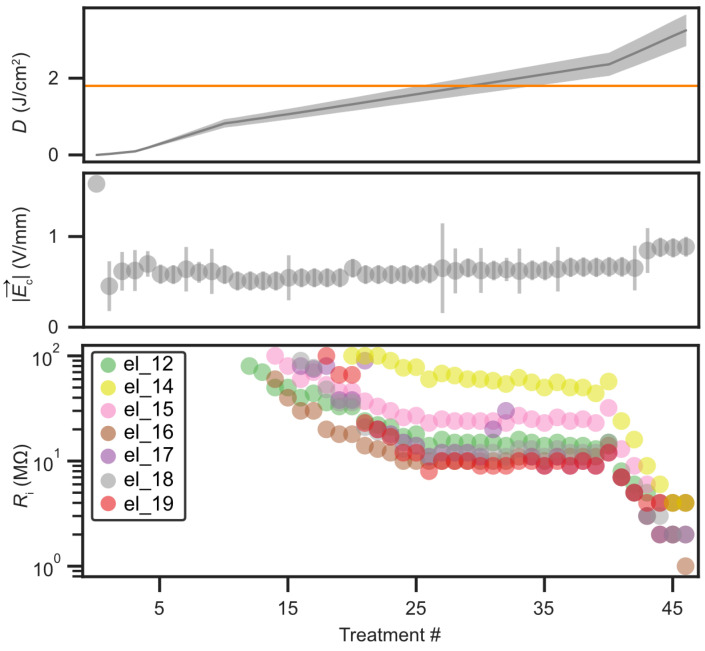
Energy dosage, compensation fields, and resistance variations during the course of Ar^+^ treatments. The top plot shows the accumulated energy dosage of $3\;J/{\mathrm{cm}}^{2}$ over 46 low–current–density (≃$0.35\;\mu A/{\mathrm{cm}}^{2}$) treatments applied to the triangular array. Each 5–15 min session corresponds to the removal of an estimated 5–6 carbon monolayers. For reference, an energy dosage of $1.8\;J/{\mathrm{cm}}^{2}$ (horizontal line), sufficient to remove approximately three monolayers, has previously resulted in a tenfold reduction in heating rate in other trap architectures [36,60,63]. The middle plot shows that, after the initial treatments, maintaining radial confinement at site $T_{2}$ required compensation fields $\left|{\vec{E}}_{c}\right|$ of about 1V/mm, which progressively decreased to ∼0.25V/mm and remained stable thereafter. The bottom plot tracks finite resistances between seven electrodes and the ground plane, all clustered near site $T_{1}$ and decreasing toward $R_{\mathrm{el}}$∼1MΩ with continued exposure.

In our main investigation, we study the impact of the Ar^+^ treatment process on the triangular-array trap. Over 46 Ar^+^ ion treatment sessions of $T_{\mathrm{treat}}≃5–15\;\min$, an accumulated dosage of $D≃3\;J/{\mathrm{cm}}^{2}$ was delivered, corresponding to an estimated removal of $n_{\mathrm{removed}}≃5–6$ carbon monolayers. The treatment was intended to remove surface contaminants and adsorbates from the gold electrodes without compromising their structural integrity. Due to its position off-axis relative to the sputtering gun’s working axis, the triangular array received a reduced current density of $J_{\mathrm{Ar}}≃0.35\;\mu A/{\mathrm{cm}}^{2}$. Figure 6 summarizes the accumulated dosage *D* and the corresponding electrical variations observed during the course of treatments. After the first treatment session, the required compensation field magnitude $\left|{\vec{E}}_{c}\right|$ at site $T_{2}$ decreased from approximately 1V/mm to 0.25V/mm and remained stable throughout subsequent sessions. Because these fields are obtained through micromotion minimization, this reduction directly reflects improved stray-field conditions near the trap center. Seven electrodes developed finite resistances, primarily near site $T_{1}$, decreasing to approximately $R_{\mathrm{el}}≃1\;M\Omega$ with increasing dosage. These finite resistances indicate the emergence of leakage paths between the affected electrodes and the ground plane.

**Figure 7:**
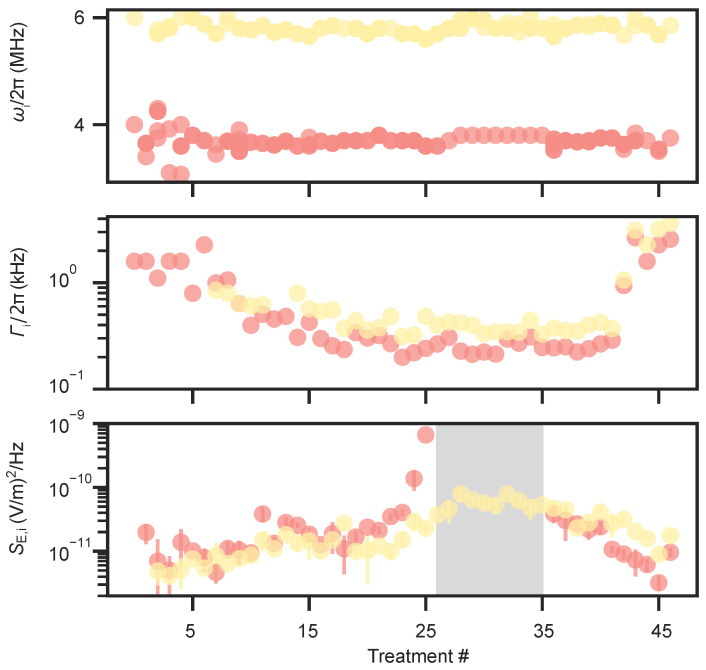
Observables using a trapped-ion sensor during the course of Ar^+^ treatments. The first plot shows the in-plane radial mode frequencies $\omega_{1}/2\pi$ and $\omega_{2}/2\pi$, stabilized at 3.7(1)MHz and 5.8(1)MHz throughout the treatments using compensation fields (red and yellow). The second plot tracks the dephasing rate $\Gamma_{i}/2\pi$ of the in-plane modes, which initially decreases and later rises to about 20kHz. The third plot shows the electric-field noise spectral density $S_{E}\left(\omega \right)$ derived from heating-rate measurements on the in-plane modes. It exhibits a non-monotonic evolution similar to that reported by Hite et al. [60], initially increasing to levels corresponding to unmeasurable heating rates (>100quanta/ms) over nine treatment sessions (gray) before declining to ≃$10^{-11}\;{\left(V/m\right)}^{2}/\mathrm{Hz}$ after 45 sessions.

To examine the influence of argon ion exposure on electric-field noise and motional coherence, we monitored the in-plane motional modes during interleaved diagnostic sessions. Figure 7 summarizes the evolution of these observables over the full treatment course. All motional modes were tracked, and the in-plane frequencies $\omega_{1}/2\pi$ and $\omega_{2}/2\pi$ remained stable at 3.7(1)MHz and 5.8(1)MHz, respectively, confirming that the ion–surface height and pseudopotential confinement remained constant throughout the treatment sequence. The motional dephasing rate $\Gamma_{i}/2\pi$ initially decreased by nearly a factor of five before gradually increasing to about 20kHz toward the end of the sequence. In contrast, the heating rate for mode $\omega_{1}$ rose steadily during the first 25 treatments, reaching unmeasurable levels of >100quanta/ms for several sessions before subsequently declining, while the heating rate for mode $\omega_{2}$ increased by an order of magnitude and later returned to levels comparable to those of $\omega_{1}$. The corresponding electric-field noise spectral density $S_{E}\left(\omega \right)$, calculated from the measured heating rates using Equation (4), exhibited a non-monotonic evolution with typical values around $10^{-11}\;{\left(V/m\right)}^{2}/\mathrm{Hz}$.

These findings highlight the narrow operational window for applying Ar^+^ sputtering to complex, multi-material electrode structures. In contrast, thicker single-layer traps can tolerate higher ion doses [18], although redeposition remains a common challenge across both architectures. Moderate treatments improved field compensation and motional coherence, whereas extended exposure promoted electrode degradation. Together, these results define the effective operational bounds under which in situ Ar^+^ cleaning can be applied to multi-layer surface-electrode traps without compromising functionality.

## 4. Discussion

By systematically isolating contributions from technical noise sources such as RF pickup, DC electrode voltage noise, and residual light leakage, we establish that all these sources of noise contribute at least two to three orders of magnitude below the observed anomalous heating rates. This suggests that the dominant contribution originates from surface-related processes intrinsic to the trap electrodes, consistent with earlier studies [17,18,60]. Our investigation reveals that in situ argon ion sputtering of multi-material surface-electrode traps can produce a nontrivial interplay between surface-induced noise, motional coherence, and electrode integrity. Consistent with established treatment studies on traps with tens-of-micrometer-thick gold top layers [18,36,60], we observe an initial increase in $S_{E}\left(\omega \right)$ even as motional dephasing rates decrease by nearly a factor of five and compensation fields drop from 1V/mm to 0.25V/mm. Simultaneously, several electrodes develop finite resistances (∼1MΩ), likely due to partial redeposition and localized structural degradation, consistent with the behavior observed in the Eurotrap material study. Post-treatment SEM/EDX analysis on the Eurotrap chip confirms substantial removal of the gold top layer alongside significant redeposition of Au and Cu near the mask edges. Extended exposure to ambient air between fabrication and vacuum operation likely contributed to additional surface oxidation and contamination [18,23,36]. These findings suggest that redeposition dynamics, surface roughening, and the top-layer diffusion collectively govern the changes in the observed noise.

These observations point to multiple concurrent microscopic mechanisms driving the changes in electric-field noise during Ar^+^ exposure. We further elaborate on three processes that likely contribute to the observed interplay between motional heating, dephasing, and electrode degradation:

1. Surface contamination and dielectric exposure: Prolonged exposure of the trap surface to ambient conditions before vacuum sealing can lead to adsorption of oxygen, hydrocarbons, and water, resulting in an unknown and spatially varying contamination load. Such uncontrolled variations are consistent with the large spread in electric-field noise observed across nominally identical traps [23]. Even after sputter cleaning, residual adsorbates may remain near the metal surface and alter local work functions. Slow surface migration of adsorbed species at ambient temperature could further modify the spatial distribution of dipoles over time, thereby influencing electric-field fluctuations [36,64]. Excessive sputtering may also thin the top metal stack and locally expose dielectric regions, which are known to host charge traps and contribute additional electric-field noise [65].
2. Surface roughening and morphological modification: The changes in heating and dephasing observed here can be qualitatively interpreted within the framework of thermally activated fluctuators. One study reported a similar non-monotonic behavior following successive ion treatments and attributed it to transient surface smoothing that increased the spatial correlation among fluctuators [23]. Within this picture, the initial removal of loosely bound contaminants shifts the activation-energy distribution of fluctuators toward higher energies, suppressing slow patch-potential drifts and improving motional coherence, while the temporary smoothing enhances high-frequency noise and thus motional heating. As sputtering continues, progressive roughening could break these correlations, which broadens the fluctuator distribution, and leads to a reduction in $S_{E}\left(\omega \right)$ accompanied by an increase in dephasing. In contrast, another study observed comparable non-monotonic heating behavior when only a few monolayers of contaminants remained on the surface, suggesting that residual adsorbates, rather than morphological evolution alone, may play a dominant role [60]. Additionally, under oblique ion-beam incidence, curvature-dependent sputter erosion can lead to the onset of nanoscale ripple patterns on the surface [66]. Such directional roughening may modify the local electric-field environment, potentially contributing to spatial variations in the field experienced by the trapped ions. Together, these experimental and theoretical insights [67] suggest that both surface morphology and adsorbate coverage jointly influence the spatial correlations of fluctuating surface dipoles, contributing to the complex, non-monotonic noise evolution observed in this work. It thus appears that surface cleaning, while often necessary, may not be sufficient to achieve low heating rates.
3. Redeposition of sputtered material: Sputtered atoms originating from both the electrode surfaces and the nearby mask, predominantly Au from the top layer and the gold-plated mask but also Pt, Ti, Al, and trace Cu, can re-adsorb non-uniformly across the trap surface. Such multi-material redeposition alters the local work function and creates microscopic potential gradients that manifest as quasi-static patch potentials and broadband electric-field noise. In addition to larger-scale coverage variations, redeposition may also form nanoscale clusters or atomic patches of metals distinct from the underlying layer (for example, small Al or Ti aggregates on Au), which can further enhance work-function contrast and local electric-field fluctuations [16]. This process is analogous to oven loading, where unintentional deposition of atomic species on trap electrodes is known to modify stray fields and increase heating [16]. We also observe the gradual formation of electrical connections between neighboring electrodes, evidenced by a measurable decrease in their ohmic resistance from the nominally open state toward values of order 0.1–1 MΩ. These conductive bridges are consistent with metallic redeposition and may introduce additional Johnson and current shot noise, as well as time-dependent resistance fluctuations when small currents flow through these connections during operation.” The redeposition efficiency and resulting electrical coupling depend strongly on the sputtering geometry, ion-incidence angle, and masking configuration, and are particularly enhanced in in situ treatments where re-adsorption paths are not actively shielded.

To harness the benefits of argon ion cleaning while mitigating adverse effects, several practical considerations arise. (I) In situ diagnostics such as optical reflectometry or residual gas analysis can be employed to monitor the onset of redeposition or over-etching during treatment, although such diagnostics alone cannot prevent these effects. (II) Careful control of sputter-beam alignment and incidence geometry, together with avoiding mesh transmission and employing a sufficiently thick top gold layer, can substantially reduce redeposition on the trap surface and nearby components. (III) Complementary gentle cleaning methods, such as UV or ozone exposure, may further reduce hydrocarbon residues without damaging metallic electrodes. In combination with these approaches, using neon ions instead of argon can suppress redeposition due to their lower sputter yield and reduced secondary scattering [68]. Additional strategies such as minimizing air exposure between fabrication and operation can improve reproducibility. Finally, the temperature dependence of sputtering effects, including cryogenic operation, remains an open topic: systematic studies of ion treatment at different substrate temperatures may help identify regimes that optimize surface cleanliness and low-noise operation [19,20,64].

Building on these practical considerations, our findings highlight the importance of continued progress in fabrication and surface treatment strategies guided by surface science and materials research, particularly for complex multilayer ion trap architectures [21,69]. Future studies could therefore focus on developing feedback-controlled sputtering protocols, incorporating complementary nondestructive cleaning techniques, and systematically exploring variations in layer composition and geometry to improve reproducibility and stability. A deeper understanding of microscopic effects such as redeposition and surface roughening will be useful for achieving both scalability and long term stability in quantum systems. The ongoing development of more complex ion traps, will continue to benefit from close collaboration between materials science and ion trapping groups. Recent reviews of quantum platforms [3,4,5,6,7,8] highlight that progress in materials science is increasingly critical for increasing coherence alongside advances in control electronics and system design. Continued exchange in experimental experience, including both successful and inconclusive results, will strengthen understanding of materials issues across communities and support steady progress toward reliable and scalable quantum technologies.

## Figures and Tables

**Table 1:** Comparison of motional distribution detection methods used in this work.

Method	Dyn. Range $\bar{n}$ (Quanta)	Req. Cooling
Sideband Thermometry	0.1–3	Ground state
Carrier	3–60	Ground state or Doppler
Doppler	100–1000	Doppler

**Table 2:** Motional heating rates due to residual light from BDx, BD, and Raman beams. Calculated expectations are provided for comparison.

Beam	Δ/2π (MHz)	$I/I_{\mathrm{sat}}$	${\dot{\bar{n}}}_{|↓\rangle }$ (Quanta/ms)	${\dot{\bar{n}}}_{|↑\rangle }$ (Quanta/ms)	${\dot{\bar{n}}}_{\mathrm{cal},|↓\rangle }$ (Quanta/ms)	${\dot{\bar{n}}}_{\mathrm{cal},|↑\rangle }$ (Quanta/ms)
BDx	0(1)	≈0.5	886(107)	16(2)	946	0.3
BD	−20	≈0.5	660(62)	11(1)	651	0.3
Raman	$+20\times 10^{3}$	≈300	1.0(2)	1.0(2)	≈0	≈0

**Table 3:** Argon ion treatment parameters for the two trap architectures.

	$V_{\mathrm{acc}}$ (kV)	$I_{\mathrm{fil}}$ (A)	$V_{\mathrm{Focus}}$ (kV)	$T_{\mathrm{Treat}}$ (min)
Eurotrap (material study)	2.0	1.8	1.6	20–45
Triangular array (main investigation)	0.5	1.8	0.5	5–15

**Table 4:** Sputter yields for Ar^+^ bombardment at normal incidence, estimated using the empirical model of Matsunami et al. [61]. These values provide approximate relative variations in sputter yield between materials and are less accurate than SRIM-based simulations [62].

Material	Thickness (Eurotrap) (nm)	Thickness (Triangle) (nm)	Yield at 2 kV (Atoms/Ion)	Yield at 0.5 kV (Atoms/Ion)
C	–	–	1.0	0.3
Au	73.7	50	3.4	1.6
Ti	30.5	25	1.0	0.5
Pt	31.3	25	2.2	1.0
Al	2400	2400	2.1	1.0
${\mathrm{SiO}}_{2}$	>9000	>9000	–	–

**Table 5:** Estimated etch rates and corresponding full-removal times for different materials under Ar^+^ bombardment at $J_{\mathrm{Ar}}=5\;\mu A/{\mathrm{cm}}^{2}$.

Material	Etch Rate (nm/s)	Time for Full Removal
Au	0.0179	69 min
Ti	0.00563	90 min
Pt	0.0104	50 min
Al	0.0110	3645 min (60.7 h)

**Figure A1:**
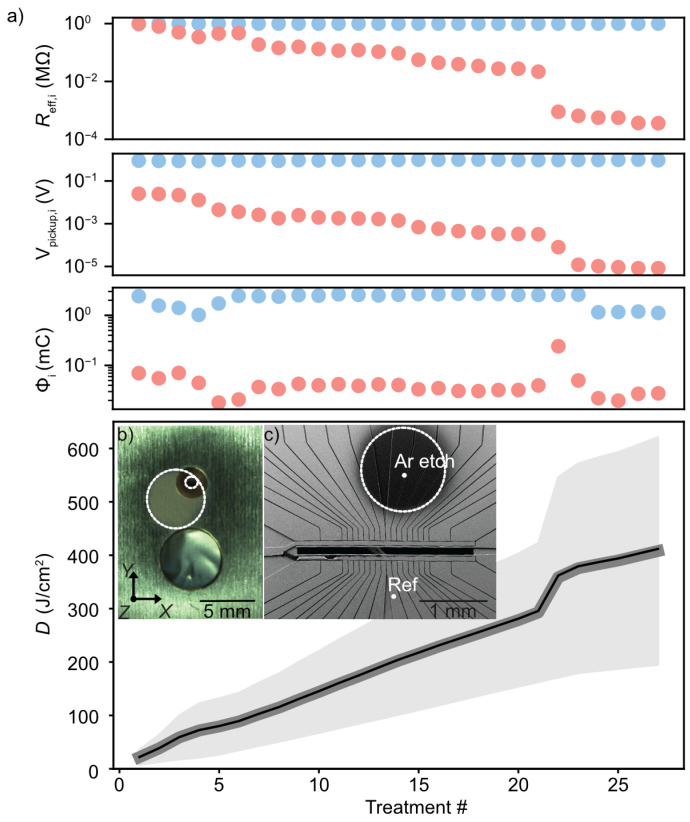
Summary of pre-investigation on the Eurotrap. (**a**) The first plot shows the evolution of effective resistance $R_{\mathrm{eff},i}$ across grouped trap electrodes (Red, $R_{\mathrm{eff},\mathrm{chip}}$) and the mask (Blue, $R_{\mathrm{eff},\mathrm{mask}}$). Each group is shunted with a ∼1MΩ resistor used to detect ion-induced pickup voltages. The second plot shows measured pickup voltages $V_{\mathrm{pickup},i}$, with mask signal stable at ∼0.94V and trap signal declining below 1mV. The third plot shows accumulated ion flux $\Phi_{i}$, with the chip receiving ∼0.045mC and the mask ∼2mC. The fourth plot shows accumulated dosage $D_{\mathrm{acc}}$, computed from $V_{\mathrm{acc}}$, $J_{\mathrm{Ar}}$, and session duration $T_{\mathrm{treat}}$. Uncertainty bounds are shown as shaded areas. (**b**) Top-view photograph of the unmounted gold-plated copper mask showing collimation and collection apertures. Discoloration marks the ion-exposed zone. (**c**) SEM/EDX image of the Eurotrap post-breakdown showing the etched region (diameter ∼1mm, edge width 0.31(1)mm) including a degraded RF electrode. EDX comparison with a nearby reference region confirms full removal of the Au/Pt/Ti stack (see Table A1) after a dosage of $D=400\left(200\right)\;J/{\mathrm{cm}}^{2}$.

## Data Availability

Data supporting the reported results can be provided upon request.

## Abbreviations

The following abbreviations are used in this manuscript:

BD	Blue Doppler (Doppler cooling beams)
BDD	Blue Doppler Detuned (Doppler cooling beams)
BDx	Blue Doppler (Resonant detection beam)
RD	Red Doppler (half of Repumping beams)
RP	Repumper (half of Repumping beams)
$B_{1},B_{3},R_{1},R_{2}$	Raman beams
TPSRT	Two-Photon Stimulated Raman Transitions
SM	Spin-Motion coupling
SBC	Sideband cooling
PI	Photo-Ionization beam
MW	Microwave
RF	Radio Frequency
DC	Direct Current
RC	Resistor and Capacitor
CMOS	Complementary metal–oxide–semiconductor
CPGA	Ceramic Pin Grid Array
PMT	Photon Multiplier Tube
SEM	Scanning Electron Microscope
EDX	Energy Dispersive X-Ray
UHV	Ultra-High Vacuum
AOM	Acousto-optic modulator
SRIM	The stopping and range of ions in matter
IR	Infra-red
ML	Monolayers
AWG	Arbitrary Waveform Generator
RMS	Root Mean Square

## Appendix A. Investigation on Eurotrap

To assess the structural and compositional effects of Ar^+^ sputtering, we performed post-treatment imaging and material analysis using Scanning Electron Microscopy (SEM) and Energy-Dispersive X-ray spectroscopy (EDX). Figure A1a summarizes the evolution of electrode resistance $R_{\mathrm{eff}}$, voltage pickup $V_{\mathrm{pickup}}$, ion flux $\Phi_{\mathrm{Trap}}$, and accumulated dosage *D* across multiple sputtering sessions. Figure A1b shows the treated mask aperture where the exposed region exhibits complete removal of gold, consistent with the estimated argon beam diameter of ∼5mm. Figure A1c displays the trap surface after treatment, with point-EDX measurements summarized in Table A1. The etched region is dominated by aluminum (93% by weight) with negligible gold, whereas the reference area retains the original Au/Ti/Pt/Al multilayer structure. These results confirm that prolonged sputtering can fully remove the gold layer and promote localized redeposition of Au/Cu near the mask edges.

**Table A1:** EDX measurement results (weight percentages). NA indicates element not detected.

Element	Au	Ti	Pt	Al	Cu	Si	O	C
Etched region	NA	1.3(1)	NA	92.7(8)	0.3(1)	0.5(1)	1.5(3)	3.7(8)
Reference region	49.4(6)	2.4(1)	13.1(5)	30.7(4)	NA	NA	NA	4.3(6)

The Eurotrap chip includes a central loading slit of approximately 100μm width for atomic vapor injection during ion loading. The metallization quality along the slit sidewalls is not characterized in detail, and incomplete coverage could lead to partially exposed dielectric regions. During Ar^+^ sputtering, the beam was not aligned to the geometric center of the trap but displaced laterally by ∼1mm, as illustrated in Figure A1b. Consequently, the ion beam did not directly strike the slit or its sidewalls. Although we cannot exclude minor contributions from charging or contamination associated with the slit surfaces, no independent measurements were conducted to quantify their influence on electric-field noise or heating rates.

SEM and EDX analyses, together with light microscopy, confirm that the main effects of in situ Ar^+^ treatment include partial removal of the gold layer, redeposition of metallic particulates (Au/Cu), and localized electrode degradation. These results guide the interpretation of electrical degradation and surface modifications discussed in Section 3.
